# ERVWE1 Reduces Hippocampal Neuron Density and Impairs Dendritic Spine Morphology through Inhibiting Wnt/JNK Non-Canonical Pathway via miR-141-3p in Schizophrenia

**DOI:** 10.3390/v15010168

**Published:** 2023-01-05

**Authors:** Wei Yao, Ping Zhou, Qiujin Yan, Xiulin Wu, Yaru Xia, Wenshi Li, Xuhang Li, Fan Zhu

**Affiliations:** 1 State Key Laboratory of Virology, Department of Medical Microbiology, School of Basic Medical Sciences, Wuhan University, Wuhan 430071, China; 2 Hubei Province Key Laboratory of Allergy & Immunology, Wuhan University, Wuhan 430071, China

**Keywords:** human endogenous retrovirus, ERVWE1, schizophrenia, miR-141-3p, Wnt5a, Arp2, dendritic spine density, dendritic spine morphology

## Abstract

Human endogenous retroviruses (HERVs) are remnants of ancestral germline infections by exogenous retroviruses. Human endogenous retroviruses W family envelope gene (HERV-W env, also called ERVWE1), located on chromosome 7q21-22, encodes an envelope glycoprotein from the HERV-W family. Mounting evidence suggests that aberrant expression of ERVWE1 involves the etiology of schizophrenia. Moreover, the genetic and morphological studies indicate that dendritic spine deficits may contribute to the onset of schizophrenia. Here, we reported that ERVWE1 changed the density and morphology of the dendritic spine through inhibiting Wingless-type (Wnt)/c-Jun N-terminal kinases (JNK) non-canonical pathway via miR-141-3p in schizophrenia. In this paper, we found elevated levels of miR-141-3p and a significant positive correlation with ERVWE1 in schizophrenia. Moreover, serum Wnt5a and actin-related protein 2 (Arp2) levels decreased and demonstrated a significant negative correlation with ERVWE1 in schizophrenia. In vitro experiments disclosed that ERVWE1 up-regulated miR-141-3p expression by interacting with transcription factor (TF) Yin Yang 1 (YY1). YY1 modulated miR-141-3p expression by binding to its promoter. The luciferase assay revealed that YY1 enhanced the promoter activity of miR-141-3p. Using the miRNA target prediction databases and luciferase reporter assays, we demonstrated that miR-141-3p targeted Wnt5a at its 3’ untranslated region (3′ UTR). Furthermore, ERVWE1 suppressed the expression of Arp2 through non-canonical pathway, Wnt5a/JNK signaling pathway. In addition, ERVWE1 inhibited Wnt5a/JNK/Arp2 signal pathway through miR-141-3p. Finally, functional assays showed that ERVWE1 induced the abnormalities in hippocampal neuron morphology and spine density through inhibiting Wnt/JNK non-canonical pathway via miR-141-3p in schizophrenia. Our findings indicated that miR-141-3p, Wnt5a, and Arp2 might be potential clinical blood-based biomarkers or therapeutic targets for schizophrenia. Our work also provided new insight into the role of ERVWE1 in schizophrenia pathogenesis.

## 1. Introduction

About 8% of the human genetic material comes from retrovirus infections that spliced their DNA into ours, known as human endogenous retroviruses (HERVs) [1,2]. Based on relatedness to exogenous genera, HERVs have been grouped into three main classes: Class I (Gammaretrovirus- and Epsilonretrovirus-like), Class II (Betaretrovirus-like), and Class III (Spumaretrovirus-like) [2,3]. Some HERVs remain unclassified because they are not similar to the reference sequence of any group. Most HERVs are named after transfer RNA (tRNA) usage. Even though HERVs are generally regarded as junk DNA for a long time, accumulating evidence suggests they play an essential role in normal physiologic processes through encoding proteins, acting as promoters/enhancers, or long noncoding RNAs (lncRNAs) [4].

Human endogenous retrovirus W family (HERV-W), originally discovered in multiple sclerosis (MS) patients and designated as MS-associated retrovirus (MSRV) [5], draws the attention of most researchers for its putative roles in several diseases, including autoimmune diseases [6], neuropsychological diseases [7], and cancers [8,9,10]. The domesticated HERV-W loci with full-length envelope gene locus on human chromosome 7q21-22, named ERVWE1, maintains a single intact open reading frame (ORF) for a complete HERV-W ENV protein (also called syncytin-1) [2]. It is expressed in the placental syncytiotrophoblast and participates in syncytium formation during placenta morphogenesis [3]. Rapidly emerging evidence indicates that increased ERVWE1 activity has been suggested in several disease, including MS [6], urothelial cell carcinoma of the bladder (UCC) [9], hepatocellular carcinoma (HCC) [10], type 1 diabetes [11], and schizophrenia [12,13,14,15,16].

Schizophrenia ranks as the top ten most common cause of disability globally, occurring in about 1% of the total population [2,13]. It imposes a tremendous economic burden not only for the individuals and their families, but also society [17]. The various schizophrenia hypotheses extend across a wide range of potential insults to nervous system, including defects in neurotransmitter systems, synapse formation, plasticity, and dendritic spines formation [18]. Postmortem examination and brain imaging techniques show that important number of schizophrenia patients have dendritic spine deficits [19]. Nevertheless, there is no study in the literature evaluating the effect of ERVWE1 on dendritic spine deficits in schizophrenia.

Actin-related protein 2 (Arp2), a major component of the actin cytoskeleton, plays an important physiological role in dendritic spine density [20], which is impaired in schizophrenia patients [21]. The Rho GTPase Rac family small GTPase 1 (RAC1) activates Arp2/3 complex-mediated actin polymerization through constitutive phosphorylation of Wiskott-Aldrich syndrome protein (WASP) at serine 483 and 484 [22,23]. Recent evidence reveals that Wingless-type family member 5A (Wnt5a) promotes Arp2/3 association at adherens junctions [24] and increases the density and maturity of dendritic spines [25]. A gene expression study suggests Wnt5a signaling is implicated in the etiology of schizophrenia [26]. The miR-141-3p, as a post-transcriptional regulator of Wnt5a expression [27], serves as a potential biomarker in Alzheimer’s disease (AD) [28].

Herein, we found the expression level of miR-141-3p was significantly up-regulated and had a positive correlation with ERVWE1 mRNA in recent-onset schizophrenia patients. Reverse transcription-quantitative polymerase chain reaction (RT-qPCR) and enzyme-linked immunosorbent assay (ELISA) showed a significant reduction of Wnt5a and Arp2 in schizophrenia compared with healthy controls. Correlation analysis showed that both Wnt5a and Arp2 had significant negative correlations with ERVWE1. A series of cell and molecular biology experiments indicated that ERVWE1 could increase the miR-141-3p level and inhibit the expression of Wnt5a through binding to transcription factor (TF) Yin Yang 1 (YY1) in SH-SY5Y cells. Additionally, miRNA target prediction and luciferase reporter assays identified that Wnt5a was a target of miR-141-3p. Further studies suggested that ERVWE1 could inhibit the expression of Wnt5a by up-regulating miR-141-3p in the human neuroblastoma cell line. An in-depth study showed that ERVWE1 down-regulated the level of Arp2 and reduced the spine density of rat hippocampal neurons. Wnt5a and the c-Jun N-terminal kinases (JNK) agonist anisomycin could relieve the inhibitory effect of ERVWE1 on neuronal morphology. Meanwhile, we also found ERVWE1 reduced dendritic spine density and dendritic branching of rat hippocampal neurons through miR-141-3p/Wnt5a.

In conclusion, this study suggested that miR-141-3p and Arp2 might be novel schizophrenia risk factors. Moreover, our results indicated that ERVWE1 reduces dendritic spine density and alters dendritic spine morphology through inhibiting Wingless-type (Wnt) non-canonical pathway in schizophrenia. Our research provided a potential diagnostic biomarker and a therapeutic target for schizophrenia. This finding also revealed important insight into the pathophysiologic role of HERV-W in schizophrenia.

## 2. Materials and Methods

### 2.1. Bioinformatics Analysis

Gene expression data of GSE35974 [29] was obtained using the R package GEOquery from the Gene Expression Omnibus (GEO) database (http://www.ncbi.nlm.nih.gov/geo/, accessed on 7 December 2018) in the National Center for Biotechnology Information (NCBI). The data was based on the GPL6244 platform (Affymetrix Human Gene 1.0 ST Array) and contained 44 cerebellum samples from schizophrenia patients and 50 cerebellum samples from healthy controls. The differentially expressed gene (DEG) analysis was performed using the limma package (http://www.bioconductor.org/, accessed on 7 December 2018). The functional enrichment of Gene ontology (GO) and Kyoto Encyclopedia of Genes and Genomes (KEGG) was conducted using the cluster profiler package, and *p* < 0.05 was considered to have statistical significance.

### 2.2. Blood Samples

This study was carried out following the principles of the Declaration of Helsinki and approved by the Ethics Committee of the Wuhan Mental Health Centre. Blood samples of patients and healthy controls, including the whole peripheral blood (18 schizophrenia patients and 25 healthy controls) for mRNA and miRNA detection, and serum samples (48 schizophrenia patients and 36 healthy controls) for ELISA analyses, were collected from the Wuhan Mental Health Centre (Wuhan, China). The patients were confirmed to meet the Diagnostic and Statistical Manual of Mental Disorders, 5th Edition (DSM-V) criteria for schizophrenia. They were diagnosed with schizophrenia for the first time, and none of them had acute infectious or psychiatric disorders. None of the healthy controls had psychiatric illnesses. There were no significant differences in gender, age, education, body mass index, and smoking status between control and schizophrenia patients. Demographic information is presented in Tables S1 and S2.

### 2.3. ELISA

The expression levels of human Wnt5a (WARNER BIO, CK-E14738, Wuhan, China), RAC1 (WARNER BIO, CK-E15408, Wuhan, China), WASP-family verprolin homologous protein 1 (WAVE1) (WARNER BIO, CK-E15629, Wuhan, China), Arp2 (WARNER BIO, N-101-107, Wuhan, China) and ERVWE1 (MEIMIAN, MM-61410H1, Wuhan, China) in serum were measured by ELISA kit according to the manufacturers’ instructions. Absorbance was measured on an ELISA plate reader (Thermo Fisher, Multiskan FC 357, Waltham, MA, USA) with a 450 nm filter and the concentrations were determined based on the standard curve.

### 2.4. Plasmid Construction and Synthesis of Mimic and Inhibitor

The plasmid of pCMV-ERVWE1 was constructed by our laboratory [12]. The full length of ERVWE1 coding sequence (CDS) was PCR amplified from pCMV-ERVWE1 plasmid and cloned into pEGFP-C1 to study the function of ERVWE1 in the rat hippocampal neurons. The coding region of the human Wnt5a gene (NM_001256105.1) was cloned into pEnter vector or pEGFP-C1 for mammalian cell expression. Wild-type (WT) Wnt5a 3’ UTR (NM_001256105.1) and mutation Wnt5a 3′ UTR (292-298: CAGUGUU to UCAGACG) luciferase reporter vectors were constructed and subcloned into pmirGLO plasmid. The promoter of miR-141-3p (Promoter-WT, NC_000012.12: 6962097-6964191), mutation miR-141-3p promoter 1 (Mut1, 6963152-6963157: GCCATA to TAAGCG), and mutation miR-141-3p promoter 2 (Mut2, 6963615-6963620: CCCATC to TATCGA) were cloned into pGL3 plasmid. The full length of the YY1 CDS (NM_003403.5) was amplified using the primer YY1-CDS-F/YY1-CDS-R (Table S3), then cloned into pCDNA3.1 plasmid and named it pCDNA3.1-YY1. The plasmid of pGL3-miR141 and pSciencer2.1-shYY1 were constructed using the same methods as described above. The target sequence of shYY1 was: 5’GCCCTCATAAAGGCTGCACAA3 ‘, which was located in NCBI reference sequence: NM_ 003403.5 (994-1014). All the recombinant plasmids were confirmed by sequencing.

The mimic, inhibitor, and the corresponding negative controls of miR-141-3p, miR-129-5p, and miR-300 were synthesized from RiboBio, Guangzhou, China (the specific sequence is listed in Table S3).

### 2.5. Cell Culture and Transfection

Human neuroblastoma cells, SH-SY5Y (ATCC, CRL-2266, Manassas, VA, USA), were cultured according to our previously published paper [16]. Sprague-Dawley (SD) rats were purchased from Wuhan University Center for Animal Experiment/A3 Laboratory. Rat hippocampal neuronal cultures were conducted as described previously [30]. The SH-SY5Y cells and rat hippocampal neurons were transfected with Lipofectamine 2000 reagents (Invitrogen, 11668019, Carlsbad, CA, USA) according to the manufacturer’s instructions.

### 2.6. RNA Extraction and Quantitative RT-qPCR

Total RNA was extracted from the cultured cells using TRIzol reagents (Invitrogen, 15596018, Carlsbad, CA, USA). The complementary DNA (cDNA) was synthesized using Moloney murine leukemia virus reverse transcriptase (Invitrogen, 28025021, Carlsbad, CA, USA) with total RNA as the template at 37 °C. RT-qPCR was carried out on the iCycler System (Bio-Rad, T100, Hercules, CA, USA) with the SYBR Green PCR master mix (Roche Diagnostics, 4913914001, Basel, Switzerland). Glyceraldehyde-3-phosphate dehydrogenase (GAPDH) was used as an internal control for quantitative comparison by the 2^−ΔΔCt^ method.

The primers for the indicated genes were designed using Primer Premier, version 5.0 (Premier Biosoft, San Francisco, CA, USA) and listed in the Table S3

### 2.7. Western Blot Analysis

Cultured cells were lysed with Mammalian Protein Extraction Reagent (ThermoFisher, 78501, Waltham, MA, USA) supplemented with phosphatases and proteases inhibitor cocktail (Abcam, ab201119, Cambridge, UK). Protein concentrations were measured using bicinchoninic acid (BCA) Protein Assay Kit (Thermo Fisher, 23225, Waltham, MA, USA). After electrophoresis on 12.5% sodium dodecyl sulfate-polyacrylamide gel electrophoresis (SDS-PAGE) gels, proteins were transferred to the polyvinylidene fluoride (PVDF) membrane (Millipore, ISEQ00010, Billerica, MA, USA) and blotted with primary antibodies. After three washes with tris buffered saline with Tween 20 (TBST), membranes were incubated with horseradish peroxidase (HRP) goat anti-rabbit IgG (Abcam, ab205718, Cambridge, UK) or HRP goat anti-mouse IgG (Abcam, ab205719, Cambridge, UK). Then, proteins were detected by enhanced chemiluminescence (ECL) Kit (Millipore, WBKLS0500, Billerica, MA, USA). The level of total protein was normalized to GAPDH (dilution 1:5000, ab8245, Abcam, Cambridge, UK), and the level of nucleoprotein were normalized to Histone H3 (dilution 1:2000, ab1791, Abcam, Cambridge, UK). The catalog number, manufacturer, and dilution of all antibodies are listed in the Table S4.

### 2.8. miRNA or mRNA Target Prediction Databases

JASPER [31], TransmiR2.0 [32], and PROMO [33] databases were used to predict the TFs that regulate the expression of miR-141-3p. The target genes of miR-141-3p were predicted using PITA [34], miRanda [35], and TargetScan [36] database. We also used PITA [34], miRanda [35], TargetScan [36], miRTarBase [37] and miRwalk [38] databases to find out the regulatory miRNA of Wnt5a.

### 2.9. TOPFlash/FOPFlash Reporter Assays

SH-SY5Y cells were transfected with ERVWE1 and negative control. Next, cells were co-transfected with the Wnt/β-catenin signaling reporter TOPFlash/FOPFlash. The reporter-driven luciferase activity was quantified by Dual-Luciferase Reporter Assay System (Promega, E1980, Madison, WI, USA). The data are represented as normalized TOPFlash/FOPFlash values.

### 2.10. Dual-Luciferase Assay

The luciferase reporter vectors containing the 3’UTR-WT or 3’UTR-Mut of Wnt5a, along with miR-141-3p mimics or negative control (NC), respectively, were co-transfected into SH-SY5Y cells. The luciferase activity was evaluated by the Dual-Luciferase Reporter Assay System (Promega, E1980, Madison, WI, USA) following the manufacturer’s instructions.

### 2.11. Co-Immunoprecipitation (Co-IP) Assay

The co-immunoprecipitation assay was performed as described previously [39]. Protein G immunoprecipitation kit (Thermo Fisher, 10007D, Waltham, MA, USA) was obtained from Thermo Scientific, rabbit Myc-tag (dilution 1:100, Abcam, ab9106, Cambridge, UK) and rabbit Flag-tag (dilution 1:100, Abcam, ab205606, Cambridge, UK) were purchased from Abcam. Western blotting was used to analyze the immunoprecipitated proteins.

### 2.12. Cellular Immunofluorescence, Confocal Microscopy, and Confocal Images Analysis and Quantification

Rat hippocampal neurons were fixed in 4% paraformaldehyde for 15 min and permeabilized with 0.25% Triton X-100 in phosphate buffered saline (PBS) for another 5 min. After washing, the cells were blocked with 5% bovine serum albumin (BSA) in PBS for 45 min. Then, the cells were incubated with diluted primary antibodies for two hours. Subsequently, samples were incubated with fluorescence-conjugated secondary antibodies for 45 min. We captured fluorescence images using Leica-LCS-SP8-STED confocal laser-scanning microscopes with 63× oil immersion objectives and an additional 2× zoom. Confocal image analysis and quantification were conducted as described before [40]. ImageJ was used for data analysis and quantification.

We transfected the ERVWE1, Wnt5a, or miR-141-3p inhibitor expression plasmid into rat hippocampal neurons at 5 days in vitro (DIV5). A sholl analysis was conducted on DIV14 and DIV21 to examine dendritic complexity and spine morphology of rat hippocampal neurons stained with microtubule associated protein 2 (MAP2, dilution 1:100, Abcam, ab5392, Cambridge, UK) and phalloidin (dilution 1:100, Abcam, ab235138, Cambridge, UK), respectively. Thus, we counted dendritic intersections across each 10 μm circle up to 150 μm distal to the soma.

### 2.13. Statistical Analysis

All data were collected from independent experiments at least three times and displayed as Mean ± standard deviation (SD). SPSS13.0 was used for the statistical analysis. For the clinical data, the median analyses and Mann-Whitney U analyses were performed. Correlation analyses were conducted using the Spearman’s rank correlation. The one-way analysis of variance (ANOVA) and Student’s t-test were used to analyze the data. The minimal level of statistical significance was set at *p* < 0.05.

## 3. Results

### 3.1. The Correlation and Consistency between miR-141-3p, Arp2, Wnt5a, and ERVWE1 in Schizophrenia Patients

**Figure 1:**
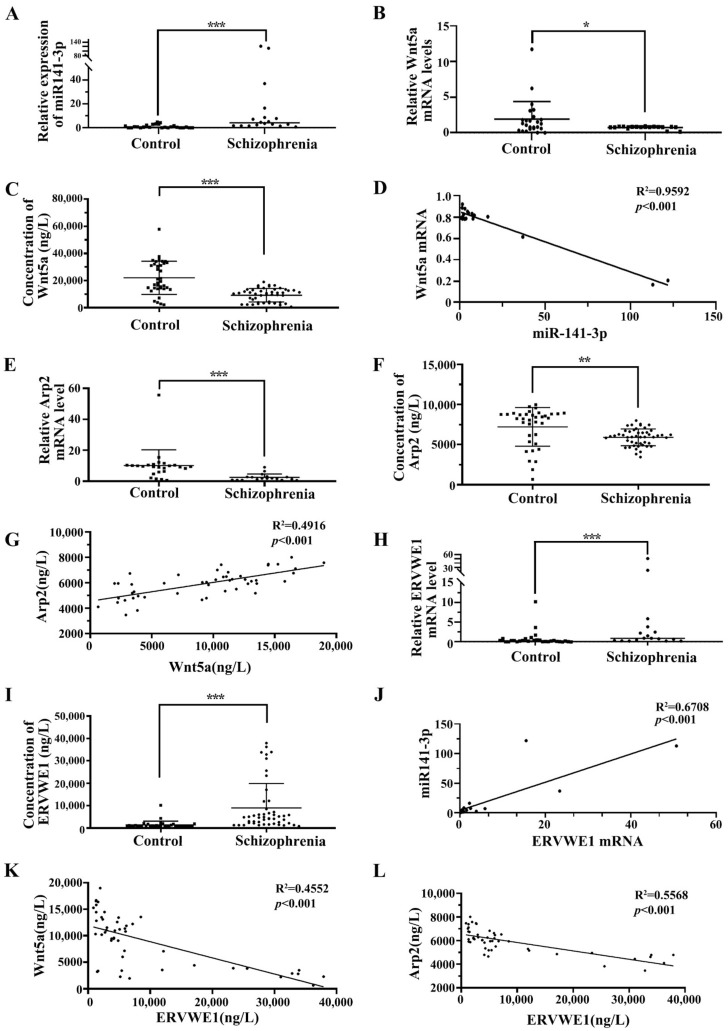
The correlation among miR-141-3p, Wnt5a, Arp2, and ERVWE1 in schizophrenia patients. (**A**) The miR-141-3p level in schizophrenia patients (n = 18) was higher than that in control groups (n = 25). (**B**,**E**,**H**) Respectively represent the mRNA levels of Wnt5a, Arp2, and ERVWE1 in schizophrenia patients (n = 18) and the control groups (n = 25) using RT-qPCR. (**C**,**F**,**I**) Respectively represent the protein expression of Wnt5a, Arp2, and ERVWE1 in the schizophrenia patients (n = 48) and in the control groups (n = 36) using ELISA. (**D**) MiR-141-3p was negatively correlated with Wnt5a mRNA in patients with schizophrenia. Where X was the miR-141-3p expression value for each sample and Y was the mRNA level for Wnt5a. (**G**) Wnt5a was positively correlated with Arp2 in patients with schizophrenia. Where X was the Wnt5a protein expression level and Y was the Arp2 protein level for each sample. (**J**) ERVWE1 was positively correlated miR-141-3p in patients with schizophrenia, where X was the ERVWE1 mRNA value and Y was the miR-141-3p level for each sample. (**K**) ERVWE1 was negatively correlated with Wnt5a in schizophrenia patients, Where X was the ERVWE1 protein value and Y was the Wnt5a protein level for each sample. (**L**) ERVWE1 was negatively correlated with Arp2 in schizophrenia patients, where X was the ERVWE1 protein value and Y was the Arp2 protein expression for each sample. *** *p* < 0.001, ** *p* < 0.05, * *p* < 0.05.

Blood-based biomarkers are regarded as a feasible option for disease identification. Till to now, no biomarker currently exists for schizophrenia. Growing evidence points out miRNA contributes to the pathogenesis of schizophrenia and can get through the blood-brain barrier [41]. Therefore, serum miRNAs have the potential to be sensitive, cost-effective blood-based biomarkers in schizophrenia [42]. In this study, qPCR results indicated that serum miR-141-3p was significantly increased in patients with recent-onset schizophrenia compared with healthy control subjects (Figure 1A). Wnt5a is one of the targets of miR-141-3p [27]. We further found decreased mRNA and protein levels of Wnt5a in schizophrenia patients (Figure 1B–C). ELISA results showed substantially lower Wnt5a expression in schizophrenia compared to healthy controls (9186.80 ± 4941.88 versus 22023.42 ± 12260.12 ng/L, respectively; *p* < 0.001) (Figure 1C and Table S5). Moreover, we also found a negative correlation between miR-141-3p and Wnt5a (*p* < 0.001, Figure 1D).

Wnt5a leads to RAC1 activation [43], which causes dissociation of the WAVE1 complex [44]. We observed that the levels of serum RAC1 (Figure S1A, Table S6) and WAVE1 (Figure S1B, Table S7) were substantially lower in schizophrenia patients than those in healthy individuals. Arp2, one of the genes downstream of the RAC1/WAVE1 signaling pathway [45,46], is significantly lower in dorsal lateral prefrontal cortex (DLPFC) layer 3 and 5 pyramidal cells in schizophrenia [21]. Our bioinformatics analysis indicated that Arp2 in postmortem human cerebellum brain was significantly lower in schizophrenia patients than in normal persons using GEO data (GSE35974) (Figure S2A–B). Blood samples of schizophrenia were collected to confirm the results of bioinformatics analysis in GEO database. Interestingly, the mRNA and protein levels of Arp2 was significantly reduced compared with the healthy control (Figure 1E–F). The median analysis indicated that the levels of serum Arp2 in the 48 schizophrenia patients were substantially decreased than that in 36 normal individuals (5909.18 ± 1041.07 ng/L versus 7216.01 ± 2408.58 ng/L respectively; *p* < 0.01) (Figure 1F, Table S8). Correlation analysis through linear regression revealed that Wnt5a protein positively associated with Arp2 protein (*p* < 0.001, Figure 1G).

Consistent with our previous study [12,13,15,16], we also observed an increased levels of ERVWE1 mRNA and protein in schizophrenia patients (Figure 1H–I, Table S9). Further analysis revealed that miR-141-3p was positively correlated with ERVWE1 mRNA level (*p* < 0.001, Figure 1J). Meanwhile, serum ERVWE1 level negatively associated with serum Wnt5a (*p* < 0.001, Figure 1K) and Arp2 levels (*p* < 0.001, Figure 1L).

The cutoff level of ERVWE1 production was set at 1996.29 ng/L, based on the levels in the healthy control sera with 95% confidence interval. Interestingly, the median analysis demonstrated that the expressions of Wnt5a and Arp2 were significantly lower in the ERVWE1 (+) persons than that in ERVWE1 (−) individuals, with the median of 9440.02 ng/L vs 15257.87ng/L (*p* < 0.05, Figure S3A), and 5937.20 ng/L vs. 7005.47 ng/L (*p* < 0.05, Figure S3B), respectively. Univariate and multivariate analyses identified miR-141-3p, Wnt5a, and Arp2 as three possible independent risk factors for schizophrenia (Table 1).

In summary, miR-141-3p, Wnt5a, and Arp2 might be novel potential blood-based biomarkers in schizophrenia.

### 3.2. ERVWE1 Up-Regulated the Expression of miR-141-3p by Combining with TF YY1

The human SH-SY5Y neuroblastoma cells can be differentiated into neuronal cells [47]. SH-SY5Y cells and hippocampal neurons have been widely used as cell models for studying the pathogenesis of schizophrenia [48,49]. Therefore, a series of experiments were conducted in SH-SY5Y cells and rat hippocampal neurons to study the role of ERVWE1 in schizophrenia.

**Figure 2:**
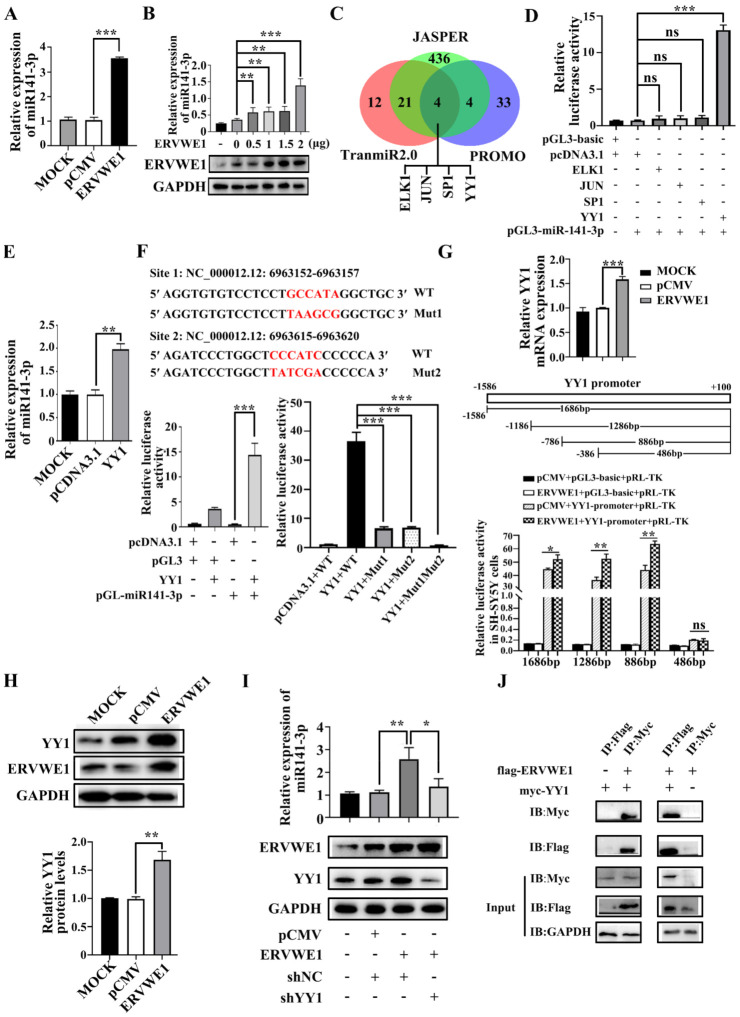
ERVWE1 promoted the expression of miR-141-3p through binding with TF YY1. (**A**) The upregulation of miR-141-3p in ERVWE1-transfected SH-SY5Y cells was validated by RT-qPCR. (**B**) The results showed that in SH-SY5Y cells containing different amounts of ERVWE1 expression plasmids (0 μg, 0.5 μg, 1 μg, 1.5 μg, 2 μg), miR-141-3p expression levels increased with the concentration of plasmid. (**C**) Prediction results showed that four TFs (ELK1, JUN, SP1, and YY1) might regulate the expression of miR-141-3p. (**D**) Analyzed the effects of various TFs on the promoter of miR-141-3p by dual-luciferase reporter assay, only YY1 could stimulate the activity of the promoter of miR-141-3p. (**E**) Increased level of miR-141-3p was detected by RT-qPCR in SH-SY5Y cells. (**F**) There were two binding sites of YY1 on the promoter of miR-141-3p, through which YY1 regulated the activity of the promoter of miR-141-3p. (**G**) ERVWE1 increased the expression of YY1 mRNA by regulating the YY1 promoter in SH-SY5Y cells; (**H**) ERVWE1 upregulated the expression level of YY1 protein. (**I**) Knockdown of YY1 substantially decreased ERVWE1-induced expression of miR-141-3p; (**J**) Specific bands were detected for Flag-tagged proteins pulled down with Myc-tagged antibody and Myc-tagged proteins pulled down with Flag-tagged antibody, which demonstrated the interaction between ERVWE1 and YY1. *** *p* < 0.001, ** *p* < 0.01, * *p* < 0.05, “ns” means no significance (*p* > 0.05).

Our clinical data suggested a significant correlation between ERVWE1 and miR-141-3p in schizophrenia. The result of cell experiment showed that ERVWE1 significantly increased the expression level of miR-141-3p (Figure 2A) in a dose-dependent manner (Figure 2B). Thus, ERVWE1 enhanced the expression of miR-141-3p.

TFs usually regulate the transcription of miRNAs [32]. Four TFs that potentially regulated the expression of miR-141-3p, namely ets-like transcription factor-1 (ELK1), jun proto-oncogene (JUN), specificity protein 1 (SP1), and YY1, were obtained from the overlap intersection of three online databases (JASPER [31], TransmiR2.0 [32], and PROMO [33]) (Figure 2C). Dual-luciferase reporter experiment suggested that YY1 was the only one that significantly enhanced miR-141-3p promoter activity (Figure 2D). Further studies showed YY1 up-regulated the expression of miR-141-3p (Figure 2E). Mutation in both sites abolished the binding of YY1 to the promoter of miR-141-3p (Figure 2F). These results indicated that these two YY1 binding sites might play a vital role in regulating miR-141-3p transcription.

Subsequently, ERVWE1 increased the mRNA level of YY1 (Figure 2G). In addition, luciferase experiments proved that ERVWE1 enhanced promoter activity of YY1 (Figure 2G). To determine the shortest sequence necessary for transcriptional regulation of YY1, we examined the activity of four promoters with truncated sequences (−1586 to +100, −1186 to +100, −786 to +100, −386 to +100). The results demonstrated that the promoter range from −786 to −386 was the shortest sequence essential for promoter activity (Figure 2G). Furthermore, ERVWE1 also up-regulated the protein level of YY1 (Figure 2H). Knockdown of YY1 substantially reduced the expression of miR-141-3p induced by ERVWE1 in SH-SY5Y cells (Figure 2I). The Co-IP assay showed that ERVWE1 directly interacted with YY1 (Figure 2J).

The above results suggested that ERVWE1 up-regulated miR-141-3p through interaction with TF YY1.

### 3.3. ERVWE1 Inhibited the Expression of Wnt5a through miR-141-3p in SH-SY5Y Cells and Rat Hippocampal Neurons

**Figure 3:**
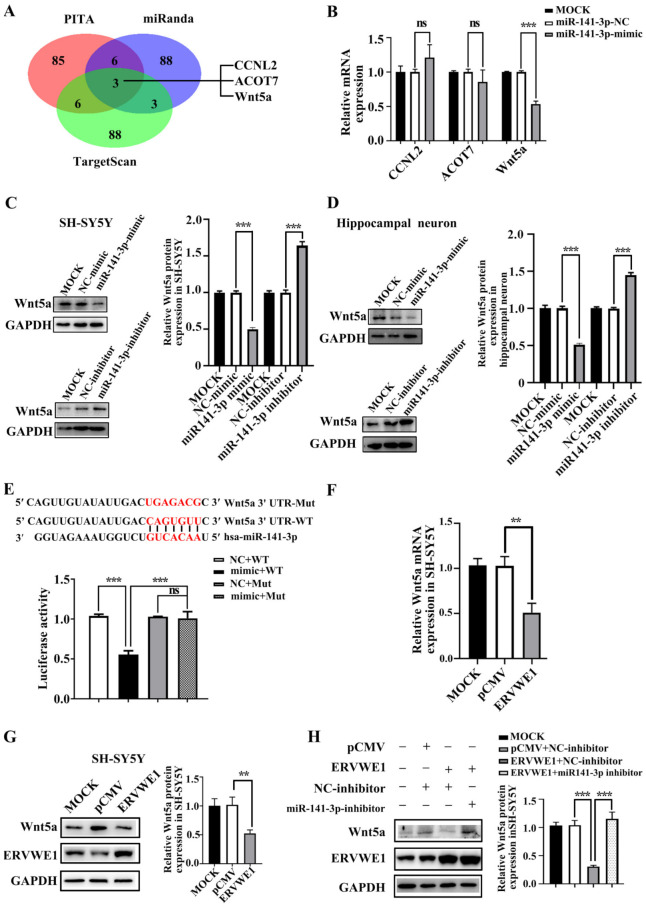
ERVWE1 reduced the expression of Wnt5a through miR-141-3p. (**A**) The prediction results showed that CCNL2, ACOT7, and Wnt5a might be the potential target genes of miR-141-3p. (**B**) RT-qPCR indicated that miR-141-3p mimic inhibited the mRNA expression of Wnt5a. (**C**,**D**) miR-141-3p mimic decreased the protein level of Wnt5a, and miR-141-3p inhibitor up-regulated the expression of Wnt5a in SH-SY5Y cells and rat hippocampal neurons. (**E**) MiR-141-3p down-regulated the expression of Wnt5a through its 3′ UTR. (**F**) The decreased level of Wnt5a was determined by RT-qPCR in SH-SY5Y cells. (**G**) ERVWE1 down-regulated the expression of Wnt5a in SH-SY5Y cells, the protein level was detected by western blot. (**H**) Knockdown of miR-141-3p substantially elevated the ERVWE1-induced protein expression of Wnt5a, the protein level was detected by western blot in SH-SY5Y cells. *** *p* < 0.001, ** *p* < 0.01, ns: *p* > 0.05.

Generally, miRNAs exert their functions by repressing their target genes [50]. We obtained three candidate targets of miR-141-3p, namely cyclin L2 (CCNL2), acyl-CoA thioesterase 7 (ACOT7), and Wnt5a, through overlapping the prediction results of three databases (PITA [34], miRanda [35], and TargetScan [36]) (Figure 3A). The results of qPCR assays showed that miR-141-3p could only inhibit the expression of Wnt5a (Figure 3B). To further confirm this, we also used PITA [34], miRanda [35], TargetScan [36] miRTarBase [37], and miRwalk [38] to find out the regulatory miRNA of Wnt5a. Three candidate miRNAs, namely miR-129-5p, miR-141-3p, and miR-300 (Figure S4A), were selected by overlapping the results from the five databases. The present results suggested that only miR-141-3p repressed the expression of Wnt5a (Figure S4B), suggesting that Wnt5a might be a target gene of miR-141-3p. The results of western blot indicated that miR-141-3p inhibited the expression of Wnt5a and elevated Wnt5a protein were observed when miR-141-3p was knocked down (Figure 3C–D). Furthermore, the TargetScan [36] database predicted that miR-141-3p binding site existed in the 3’ UTR of Wnt5a (CAGUGUU, positions 292-298). Site-directed mutagenesis reversed the effect of miR-141-3p on Wnt5a, indicating that miR-141-3p regulated Wnt5a through its 3’ UTR region (Figure 3E). In summary, Wnt5a was a target of miR-141-3p.

Our clinical data revealed a negative correlation between Wnt5a and ERVWE1 in schizophrenia patients. Intriguingly, we discovered that ERVWE1 significantly decreased the mRNA and protein level of Wnt5a in SH-SY5Y cells and rat hippocampal neurons (Figure 3F–G and Figure S4C,D). Additionally, the miR-141-3p inhibitor markedly impaired the decreased protein expression of Wnt5a induced by ERVWE1 in SH-SY5Y cells (Figure 3H), suggesting that ERVWE1 restrained the expression of Wnt5a through by miR-141-3p.

### 3.4. ERVWE1 Inhibits the Expression of Arp2 via Inhibiting Wnt/JNK Non-Canonical Pathway, neither Wnt Canonical nor Wnt/Calcium Non-Canonical

**Figure 4:**
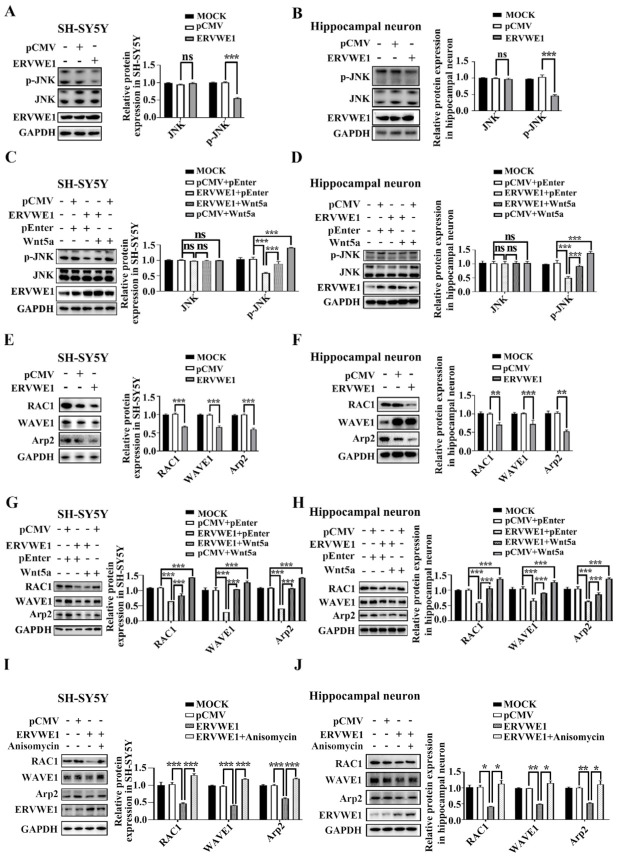
ERVWE1 lessen the expression of Arp2 via JNK-mediated non-canonical signaling pathway. (**A**,**B**) ERVWE1 down-regulated the protein levels of p-JNK but had no effects on the level of total-JNK in SH-SY5Y cells and rat hippocampal neurons. (**C**,**D**) Elevated level of Wnt5a could increase the ERVWE1-induced protein expression of p-JNK in SH-SY5Y cells and rat hippocampal neurons. (**E**,**F**) ERVWE1 down-regulated the protein level of RAC1, WAVE1, and Arp2 in SH-SY5Y cells and rat hippocampal neurons. (**G**,**H**) Elevated of Wnt5a could increase the ERVWE1-induced protein level of RAC1, WAVE1, and Arp2 in SH-SY5Y cells and rat hippocampal neurons. (**I**,**J**) Anisomycin elevated the ERVWE1-induced protein level of RAC1, WAVE1, and Arp2 in SH-SY5Y cells and rat hippocampal neurons. *** *p* < 0.001, ** *p* < 0.01, * *p* < 0.05, ns: *p* > 0.05.

We found that ERVWE1 decreased the expression of Wnt5a. Reduced Wnt expression leads to abnormal activation of the Wnt signaling pathway [51]. Thus, we examined whether ERVWE1 caused aberrant Wnt signaling in SH-SY5Y cells and rat hippocampal neurons. The results indicated that ERVWE1 did not affect the levels of total JNK, but decreased the phosphorylation of JNK (Figure 4A–B), suggesting that ERVWE1 suppressed the Wnt/JNK non-canonical pathway. Further experiments found that Wnt5a could alleviate the inhibition of active *p*-JNK by ERVWE1 (Figure 4C–D).

However, the level of β-catenin and phosphorylation of calcium/calmodulin dependent protein kinase II alpha (CAMK2A) remained unchanged in SH-SY5Y cells and rat hippocampal neurons (Figure S5A–B), indicating that ERVWE1 had no effect on neither Wnt canonical pathway nor Wnt/Ca^2+^ non-canonical signaling pathway. TOPflash/FOPflash reporter system also authenticated that ERVWE1 failed to affect the transcriptional activity of β-catenin (Figure S5C), implying that ERVWE1 had no effect on the Wnt canonical pathway. Upregulating Wnt5a did not alter the effect of ERVWE1 on β-catenin, CAMK2A and *p*-CAMK2A (Figure S5D–E). These results indicated that ERVWE1 inhibited the Wnt signal transduction through Wnt /JNK non-canonical pathway, neither Wnt/β-catenin nor Wnt/Ca^2+^.

Our clinical results demonstrated a negative correlation between ERVWE1 and Arp2 in schizophrenia patients. Here, we found that ERVWE1 significantly downregulated the protein expression of RAC1, WAVE1, and Arp2 in SH-SY5Y cells and rat hippocampal neurons (Figure 4E–F). However, actin related protein 3 (Arp3), one of seven subunits of the Arp2/3 complex, remained unchanged (Figure S5F).

Wnt5a activates Arp2 through RAC1 and WAVE1 [23,25,43,52,53]. Our clinical data implied that Wnt5a was positively correlated with Arp2. We further assessed the possible role of Wnt5a in ERVWE1-mediated Arp2 expression. The results showed that Wnt5a increased the level of RAC1, WAVE1, and Arp2 (Figure 4G–H). Further studies indicated that Wnt5a (Figure 4G–H) and a JNK activator anisomycin (Figure 4I–J) relieved the inhibition of RAC1, WAVE1, and Arp2 induced by ERVWE1. But there was no effect on Arp3 (Figure S5G–H). Together, these results suggested that ERVWE1 regulated the RAC1, WAVE1, and Arp2 through the suppression of Wnt5a /JNK non-canonical pathway.

In summary, these findings indicated that ERVWE1 suppressed Arp2 via Wnt5a/JNK non-canonical pathway, not Wnt/calcium non-canonical pathways or Wnt canonical pathway.

### 3.5. ERVWE1 Inhibited the Wnt/JNK Non-Canonical Signal Pathway Depending on miR-141-3p

**Figure 5:**
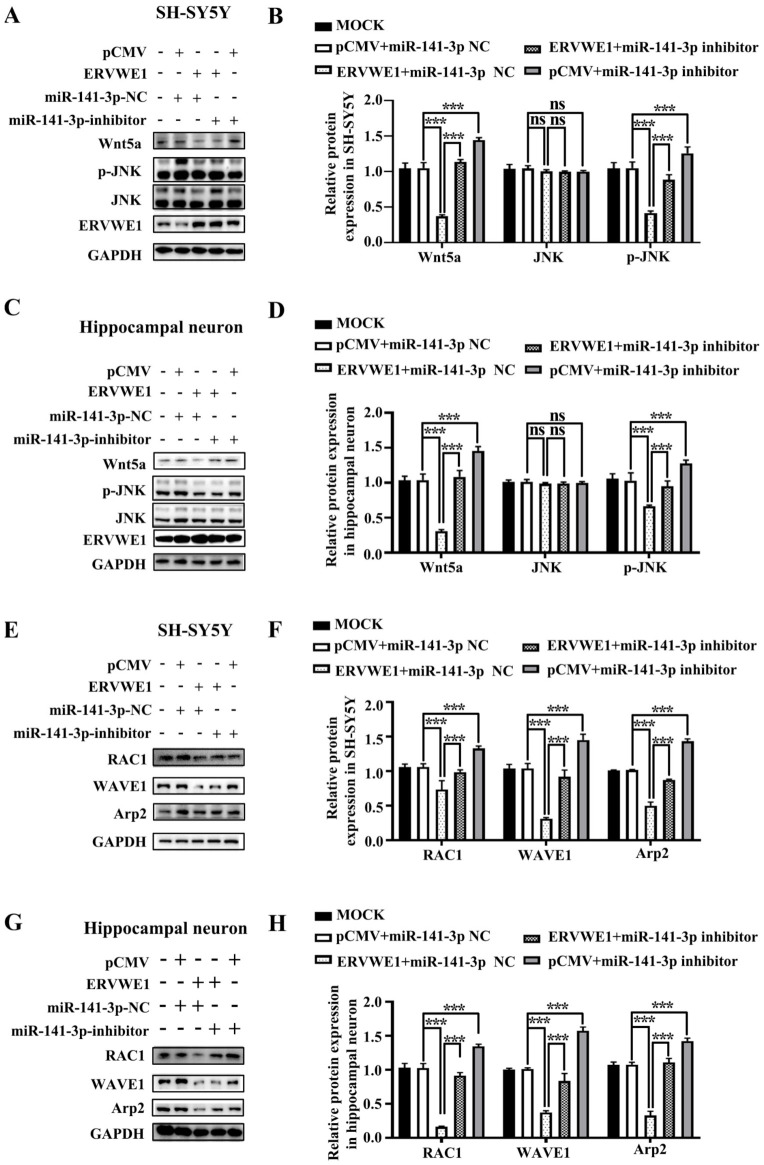
MiR-141-3p mediated the inhibition of the JNK non-canonical signal pathway induced by ERVWE1. (**A**,**B**) Western blot results showed that knockdown of miR-141-3p substantially elevated the ERVWE1-induced protein expression of p-JNK in SH-SY5Y cells. (**C**,**D**) In rat hippocampal neurons, miR-141-3p knockdown resulted in an increase in p-JNK proteins induced by ERVWE1. (**E**,**F**) In SH-SY5Y cells, miR-141-3p knockdown significantly increased the expression of RAC1, WAVE1, and Arp2 induced by ERVWE1. (**G**,**H**) The knockdown of miR-141-3p significantly increased the expression of RAC1, WAVE1, and Arp2 in rat hippocampal neurons induced by ERVWE1. *** *p* < 0.001, ns: *p* > 0.05.

Our above results demonstrated that Wnt5a was the target of miR-141-3p and ERVWE1 significantly inhibited the Wnt non-canonical signal. Thus, we analyzed the possible role of miR-141-3p in ERVWE1-induced inhibition of Wnt/JNK signaling pathway. The results manifested that miR-141-3p inhibitor recovered the level of Wnt5a and phosphorylated JNK (Figure 5A–D). We also discovered that miR-141-3p inhibitor reversed the decreased expression of Wnt signal downstream genes including RAC1, WAVE1, and Arp2 (Figure 5E–H).

ERVWE1 did not activate Wnt canonical and Wnt/calcium non-canonical signaling pathways. Here, our studies also revealed that miR-141-3p inhibitor had no effect on the expression of β-catenin, *p*-CAMK2A (Figure S6A–B), further proving that ERVWE1 did not activate Wnt canonical and Wnt/calcium non-canonical signaling pathways.

Together these observations indicated that the inhibition effect of ERVWE1 on the Wnt/JNK non-canonical signal pathway was mediated by miR-141-3p.

### 3.6. ERVWE1 Caused Significant Changes in Dendritic Spine Density and Dendritic Complexity through miR-141-3p/Wnt5a

**Figure 6:**
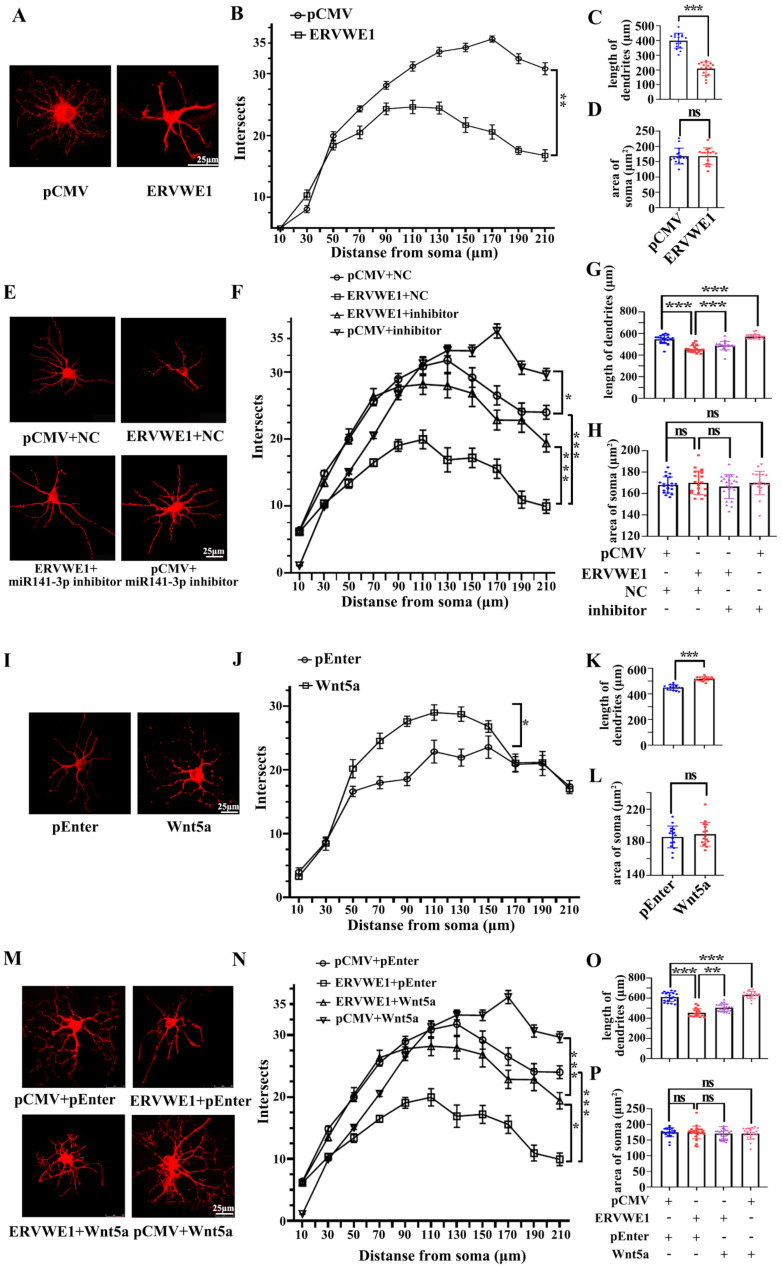
ERVWE1 changed the density of dendritic spine through miR-141-3p in rat hippocampal neurons. (**A**,**E**,**I**,**M**) Primary rat hippocampal neurons were transfected with ERVWE1, ERVWE1 and miR-141-3p inhibitor, Wnt5a, ERVWE1 and Wnt5a, then imaged at DIV14 and were immunostained with MAP2. High-magnification images were obtained by confocal microscopy. (**B**,**F**,**J**,**N**) The sholl analysis of dendrites from rat hippocampal neurons. (**C**,**G**,**K**,**O**) The total length of dendrites was measured by ImageJ software. (**D**,**H**,**L**,**P**) The soma area was measured by ImageJ software. *** *p* < 0.001, ** *p* < 0.01, * *p* < 0.05, ns: *p* > 0.05.

The morphology and density of dendritic spine are crucial for synaptic plasticity. Accumulating evidence has indicated abnormal synaptic plasticity and cognitive impairments in schizophrenia [54]. Reduced dendritic spines density is the more likely source of the deficit in schizophrenia [55]. The sholl analysis revealed that ERVWE1 had a significant effect on dendritic complexities in rat hippocampal neurons (Figure 6A). ERVWE1 significantly reduced the number of dendritic intersections crossing between 50μm to 210 μm from the center of the cell soma (Figure 6B). ERVWE1 also caused a significant decrease in total dendrite length in rat hippocampal neurons (Figure 6C), but had no effect on neuron soma size in the hippocampus (Figure 6D). Subsequent experiments investigated that miR-141-3p inhibitor relieved the changes in dendritic complexity (Figure 6E), total spine density deficiency (Figure 6F), and length (Figure 6G) caused by ERVWE1. However, there was no effect on the area of rat hippocampal neuron cell bodies (Figure 6H). Wnt5a substantially increased dendritic complexity (Figure 6I-J) and the total length of dendrites (Figure 6K), but had no effect on hippocampal neuron soma size (Figure 6L). Similarly, Wnt5a also alleviated the decrease in dendrite complexity (Figure 6M), the inhibition of the complexity of dendritic spinal branching (Figure 6M–N), and total dendritic length (Figure 6O) caused by ERVWE1, but not on the size of neuronal cell bodies (Figure 6P). These results suggested that ERVWE1 attenuated dendritic complexity and enormously decreased total dendritic length through miR-141-3p/Wnt5a.

### 3.7. ERVWE1 Regulated Dendritic Spine Morphology through miR-141-3p/Wnt5a

**Figure 7:**
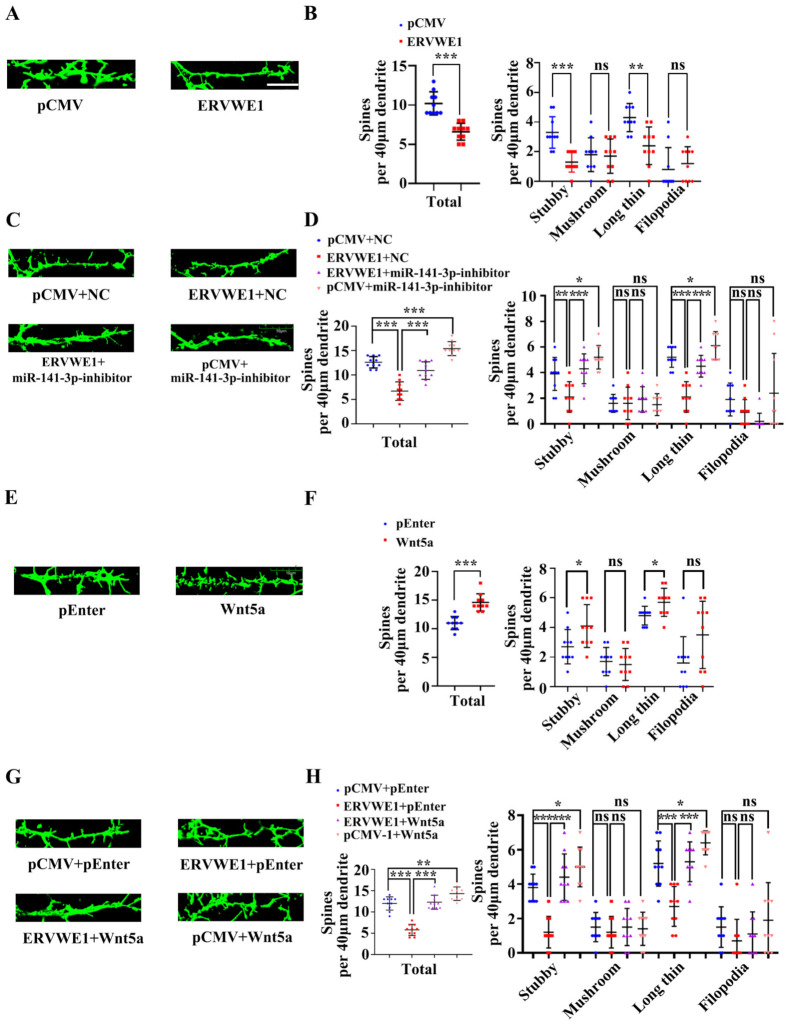
ERVWE1 altered dendritic spine morphology through miR-141-3p/Wnt5a in rat hippocampal neurons. (**A**) Fluorescence micrographs revealed that a decreased number of spines in rat hippocampal neurons with ERVWE1. (**B**) The statistics for the total and classified spine numbers per 40 μm dendrite for A, quantification of spine morphology reveals a decreased number of stubby and long-thin spines. (**C**) The number of spines might be recovered by miR-141-3p by fluorescence images of neurons labeled with phalloidin. (**D**) The statistics for the total and classified number of spines per 40 μm dendrite were calculated for C. (**E**) Fluorescence micrographs revealed that an increased number of spines in rat hippocampal neurons with Wnt5a. (**F**) The statistics for the total and classified number of spines per 40 μm dendrite were calculated for E, quantification of spine morphology reveals an increased number of stubby and long-thin spines. (**G**) A phalloidin-labeled neuron showed that Wnt5a might restore spine number. (**H**) The statistics for the total and classified number of spines per 40 μm dendrite were calculated for G. *** *p* < 0.001, ** *p* < 0.01, * *p* < 0.05, ns: *p* > 0.05.

Further analysis revealed that ERVWE1 had a significant effect on the pattern of dendritic branching at DIV21 in rat hippocampal neurons (Figure 7A). The number of dendritic spines along a 40 μm dendrite was dramatically reduced (Figure 7B). Spines are classified as stubby, mushroom, long-thin, or filopodia spines as described [56]. An in-depth analysis suggested that ERVWE1 led to a modest but significant decrease in the number of stubby and long-thin spines, but had no effects on mushroom and filopodia spines (Figure 7B). Later studies found that miR-141-3p restored the reduced number of spines along a 40 μm dendrite (Figure 7C), stubby and long-thin spines due to ERVWE1 (Figure 7D), but had no effect on mushroom and filopodia dendritic spines (Figure 7D).

Wnt5a increased the total number of spines along a 40 μm dendrite length in rat hippocampal neurons, among which dendritic spines were mainly stubby and long-thin dendritic spines, but have no effect on mushroom and filopodia dendritic spines (Figure 7E,F). Likewise, Wnt5a also reversed the reduction in the number of hippocampal spines along a 40 μm dendrite (Figure 7G), stubby and long-thin spines (Figure 7H), but no on mushroom and filopodia dendritic spines (Figure 7H). These advised that ERVWE1 influenced dendritic spine morphology through miR-141-3p/Wnt5a.

In conclusion, these findings denoted that ERVWE1 alters hippocampal neuron density and dendritic spine morphology via miR-141-3p/Wnt5a.

## 4. Discussion

In general, HERVs localize in the heterochromatin and are silent in mammalian somatic tissues to prevent their uncontrolled expression. But in some condition, certain ERVs are activated by external (ultraviolet (UV) [57], smoking [57], infections [58], and chemical drugs [59]), internal (morphogens, hormones, cytokines, and gene mutation), and epigenetic factors (DNA methylation and histone modification), which will ultimately cause diseases, MS [60], cancer [10], and schizophrenia [7,12,13,15,16]. HERVs contribute to development of disorders in various ways, such as assembling viral particles [2], acting as a promoter to regulate the expression of nearby cellular genes [8], or expressing viral proteins to regulate host cell signaling networks [12,13,15,16,61,62,63,64,65]. Our studies suggest an abnormal expression of ERVWE1 in schizophrenia [12,13,15,16]. Further studies indicate that ERVWE1 can directly activate Na^+^ [16], Ca^2+^ [14,61] or K^+^ [14,62] ion channels, which are widely expressed in the central nervous system and implicate in the pathophysiology of schizophrenia [14,16,61,62]. Moreover, we find that ERVWE1 results in neuroinflammation, which plays a major role in the neurobiology of schizophrenia [2,13], through increasing the production of nitric oxide (NO) [65], triggering a strong Cytotoxic T lymphocyte (CTL) activity [63], or increasing inflammatory markers including C-reactive protein (CRP) [13], interleukin 6 (IL-6) [13], tumour necrosis factor alpha (TNF-α) [64], and interleukin 10 (IL-10) [64]. Additionally, our research authenticates that ERVWE1 regulates the expression of brain derived neurotrophic factor (BDNF), one of schizophrenia risk gene, through glycogen synthase kinase 3β (GSK3β) phosphorylation at Ser9 [66]. Interestingly, we also discover that ERVWE1 contributes to mitochondrial dysfunction in neuron [15] and triggers abnormal dopaminergic neuron process through dopamine receptor D2 (DRD2) [16]. Together, ERVWE1 may contribute to the etiology of schizophrenia through a series of biological processes. In this study, we showed that miR-141-3p might serve as a predictive biomarker or therapeutic target for schizophrenia. Further studies disclosed the ERVWE1 effect of inhibition on the morphology and dendritic spine density of hippocampal neurons by suppressing the Wnt/JNK non-canonical pathway through miR-141-3p in schizophrenia.

Up to now, diagnosis of schizophrenia is still based on a full psychiatric evaluation, medical history assessment, and physical exam [67]. No diagnostic biomarker has been identified in schizophrenia [41]. Blood-based biomarkers may help clinicians make the right diagnosis [67]. Recent studies discover that the etiopathology of schizophrenia is associated with altered expression or function of miRNAs [41]. There are some good evidences that miRNAs are easily accessible blood-based biomarkers for detecting a variety of complex diseases. Studies have shown that serum miR-25 is used as a novel biomarker to assist early diagnosis of pancreatic cancer in China [68]. A lot of evidence points out that miR-141-3p, as one of the most abundant exosomal miRNA exclusively present in the inflammatory response, may be a diagnostic and prognostic blood-based biomarker in several diseases, including cancers [69] and neuropsychical disease [28]. Recent studies illustrate that plasma exosomal miR-141-3p, significant differences in the AD group, has high diagnostic value in AD [28]. Several researchers report that, serum miR-141-3p, reduced in the early stage of Parkinson’s disease (PD) patients, may represent a novel biomarker for the early detection of PD [70]. Our data suggested that serum miR-141-3p, increased in schizophrenia, might be a novel potential blood-based biomarker and risk factor for schizophrenia.

Plasma proteins also yield a blood-based screening tool for helping diagnose diseases [71]. There is now strong evidence supporting potential blood-based protein biomarkers predict progression of schizophrenia [72]. Our data displayed Arp2 as a possible independent risk factor for schizophrenia. Recent studies have suggested that Arp2 [21] and RAC1 [73] are significantly lower in schizophrenia, similar to our findings. In conclusion, Arp2, associated with abnormalities in dendritic spines [74], might serve as a potential protein biomarker for schizophrenia.

Several studies suggest an abnormal Wnt gene expression in schizophrenia. It has been reported that plasma levels of soluble dickkopf 1 (DKK-1) and sclerostin are significantly lower in schizophrenia than in healthy controls [75]. Nuclear factor of activated T cells 3 (NFATc3), a transcription factor associated with the non-canonical Wnt/Ca^2+^ pathway, and frizzled class receptor 7 (FZD7), a transcription factor that may stimulate non-canonical Wnt signaling, are significantly increased in the whole blood of schizophrenia patients [75]. Our findings evaluated the plasma levels of Wnt5a in schizophrenia patients. The results also underscored the fact that secreted Wnt modulators might be potential biomarkers for schizophrenia and likely targets for the development of novel therapeutics [75].

Our clinical data demonstrated a positive correlation between miR-141-3p and ERVWE1 in schizophrenia patients. Subsequent cytological experiments confirmed that ERVWE1 promoted the expression of miR-141-3p. MiR-141-3p is one of widely studied miRNAs in diseases. Quite a few reports show that dysregulation of miR-141-3p predicts poor outcomes in several cancers, such as HCC, glioma, colorectal cancer, prostate cancer, and etc [76]. Furthermore, miR-141-3p induces apoptosis, oxidative stress, and mitochondrial dysfunction in vitro model of PD [77]. Our in-depth research showed that miR-141-3p was involved in the nerve cell damage caused by ERVWE1, indicating that miR-141-3p took part in the pathological process of many diseases, including schizophrenia.

The interaction between TFs and miRNAs plays a critical role in the regulation of biological networks. TFs act directly on miRNA promoters [32]. YY1 is a zinc finger protein that activates gene expression depending on interacting partners, promoter context and chromatin structure [78]. YY1 also plays critical roles in the development of the mammalian nervous system. It mediates the transcription of neuronal survival genes, as well as neuronal differentiation, specification and migration [79]. Any impairment of functional YY1 may cause neuronal death [79], suggesting the pathogenic roles of YY1 in neuropyschological diseases. There are almost no studies of the role of YY1 in schizophrenia. Only one study reports that the missense schizophrenia risk variant rs1801311 (located in the 1st exon of NDUFA6 gene) can disrupt the binding of YY1, TAF1, and POLR2A [80]. Our observation indicated that YY1 activated the expression of miR-141-3p, representing that it might play a momentous role in the pathogenesis of schizophrenia.

MiRNAs function in the post-transcriptional regulation of gene expression [50]. Recent research shows that miR-141-3p reduces cell migration and proliferation by targeting Wnt5a in atherosclerosis model [27]. Our experiments also confirmed that wnt5a was a target gene of miR-141-3p. In vitro and vivo models indicate that Wnt5a modulates dendritic spine dynamics [25] and promotes postsynaptic development [81] in hippocampal neurons.

The aberrant expression of Wnt5a is reportedly involved in the progression of several neuropsychiatric disorders. Wnt5a plays a key role in spinal dendritic spine remodeling in neuropathic and inflammatory pain models [82]. Another report mentions that Wnt5a alters neuron cholesterol metabolism and alleviates cognitive impairment in a progressive PD model [83]. Recent studies have demonstrated that aberrant Wnt5a is associated with the pathogenesis of schizophrenia [26,75]. We found that ERVWE1 downregulated Wnt5a and Wnt5a could reversed the neuron damage caused by the ERVWE1 and miR-141-3p, denoting that Wnt5a might improve motor and cognitive impairment during the pathogenesis of schizophrenia.

There are three Wnt signaling pathways, including the canonical Wnt pathway, the noncanonical Wnt/JNK pathway, and the noncanonical Wnt/Ca^2+^ pathway [84]. Wnt5a is one of the non-canonical Wnt ligands. In this paper, we found Wnt5a activated noncanonical Wnt/JNK signaling, not Wnt/β-catenin or Wnt/Ca^2+^. Wnt signaling network may be of key relevance for the pathogenesis of neuropsychiatric disorders [85]. An abnormal Wnt signaling pathway is associated with the pathophysiology of various neurological and neuropsychiatric disorders, including AD [86], bipolar disorder [75], and schizophrenia [26]. Increasing attention is focused on the role of Wnt signaling pathways in schizophrenia [86]. Aberrant Wnt signaling has been implicated in the pathophysiology of schizophrenia [26,75]. We interestingly find that ERVWE1 inhibited the Wnt5a signal transduction through Wnt/JNK non-canonical pathway, implying that ERVWE1 might cause abnormal nerve cell function by inhibiting the Wnt signaling pathway, eventually leading to schizophrenia.

Several studies indicate that Wnt5a induces the activation of RAC1, a member of the Rho family of GTPases [43]. RAC1, a cytoskeleton regulation protein that amends cell adhesion, morphology, and movement, regulates spine dynamics dendritic, spine morphology, dendrite development, and spine formation as well as structural plasticity of mature spines [87]. Abnormal RAC1 is responsible for delayed neuronal death, neuronal degeneration, and cognitive dysfunction in hippocampal neurons and contributes to different neurodevelopmental disorders [88], such as schizophrenia [73]. Postmortem studies indicate that RAC1 is significantly decreased in the gray matter of subjects with schizophrenia [73]. RAC1 contributes to the pathogenic mechanism of schizophrenia as the downstream signaling central molecule of several schizophrenia risk genes [88], including disrupted-in-schizophrenia 1 (DISC1) [89] and brain-derived neurotrophic factor (BDNF) [88], which regulate neuroplasticity and neural connectivity. Results from our previous studies demonstrate that ERVWE1 regulates the levels of BDNF [12] and DISC1 [61] in schizophrenia. Here, we further discovered that ERVWE1 led to great variability in RAC1 levels, indicating a role of ERVWE1 in the pathogenesis of schizophrenia via RAC1.

WAVE complex is one of two main downstream effector pathways of RAC1 [53]. As a member of the WASP family, WAVE1, a key regulator in the formation of the filamentous actin cytoskeleton, controls neuronal activity-induced mitochondrial distribution in dendritic spines and regulates spine morphogenesis and neuronal polarization [90]. Abnormalities in WAVE1 contributes to the susceptibility of neuropsychiatric disorders [91]. Decrease WAVE1 mRNA is observed in human AD brains and reduction WAVE1 restores memory deficits in AD mouse model [92]. However, there is no report on the role of WAVE1 in schizophrenia. Our study showed that ERVWE1 inhibited the activity of RAC1 and WAVE1 through Wnt5a in neurons, suggesting that ERVWE1 triggered schizophrenia by regulating RAC1 and WAVE1 in schizophrenia.

WAVE1 can stimulate the Arp2/3 complex [93]. Arp2/3, one of three known actin nucleators in eukaryotes, is directly involved in functional maturation of dendritic spines [20]. In addition, down-regulation of the Arp2/3 complex destroys spine structure and leads to their loss in schizophrenia [21]. Likewise, disruption of Arp2/3 causes structural plasticity and dendritic spine loss in forebrain excitatory neurons in vivo [74]. Haloperidol antipsychotic treatment changes the behavior of Arp2/3 mutant mice [94], suggesting a link between Arp2/3 complex and psychiatric disorders like schizophrenia. A significant reduction is found in transcript levels of Arp2/3 in DLPFC layer 3 and 5 pyramidal cells of schizophrenia patients [21]. Arp2 is one of the subunits of the Arp2/3 complex. Synaptic Arp2 level is reduced in Down syndrome [95], AD [95], and schizophrenia [21]. In this paper, ERVWE1 down-regulated the expression of Arp2 through Wnt/JNK signaling pathway but had no effect on Arp3, mentioning that ERVWE1 might change dendritic spines in neurons, subsequent leading to neuronal abnormalities in schizophrenia.

Wnt5a, RAC1, WAVE1 and Arp2 are critical regulators in the development of dendritic spines [20,25,87,96]. Wnt5a, expressed early in neuronal development, increases dendritic spine density, regulates dendritic spine formation, and stimulates dendritic spine morphogenesis [25]. RAC1 influence the morphological maturation of dendritic spines [87]. Loss of WAVE1 induces a decrease in mature dendritic spines [96]. The Arp2/3 complex is essential in functional maturation of dendritic spines [20]. Dendritic spines, small membranous protrusion from a neuron’s dendrite, serve as the postsynaptic platform and help transmit electrical signals to the neuron’s cell body. Dendritic spine morphogenesis is associated with the formation of learning and memory [19]. Changes in dendritic spine number and morphology results in remodeling of connectivity within neuronal circuits [97]. Postmortem studies in schizophrenia suggest that reduced density of dendritic spines is one of the hallmark neuropathological alterations [97]. Gray matter loss in schizophrenia is due to a reduction in spine density [19].In our study, ERVWE1 led to decreased dendritic spine density, which might be one of the pathogenesis mechanisms of schizophrenia.

Dendritic spines receive synaptic contacts that are necessary in the processes of learning and memory. Changes in dendritic spine morphology parallels cognitive decline [97]. Altered dendritic spine morphology is also a hallmark of neuropsychological disorders [97]. Studies of human neuropathologies point out that dendritic spine dysmorphogenesis links to cognitive deficits [97]. Neuropathological studies display abnormal dendritic spine morphology in schizophrenia [97]. Postmortem notes morphological changes in dendritic spines in brains of patients with schizophrenia and also in animal models of schizophrenia [19]. These changes can lead to altered connectivity and coherence within and between brain regions, which may underlie the emergence of behavior alterations [98]. Mounting evidences demonstrate that dendritic spine morphological abnormalities may contribute to the cognitive disorder of schizophrenia [19], suggesting that morphological changes in dendritic spines may be related to memory and cognitive impairment in schizophrenia. Here, we found that ERVWE1 contributed to neuronal morphological changes, which might help understand the etiology and pathophysiology of schizophrenia.

## 5. Conclusions

From the above, we implicated decreased Wnt5a/JNK non-canonical pathway as a mechanism of abnormal dendritic spines morphogenesis induced by ERVWE1, which would ultimately lead to schizophrenia.

**Figure 8:**
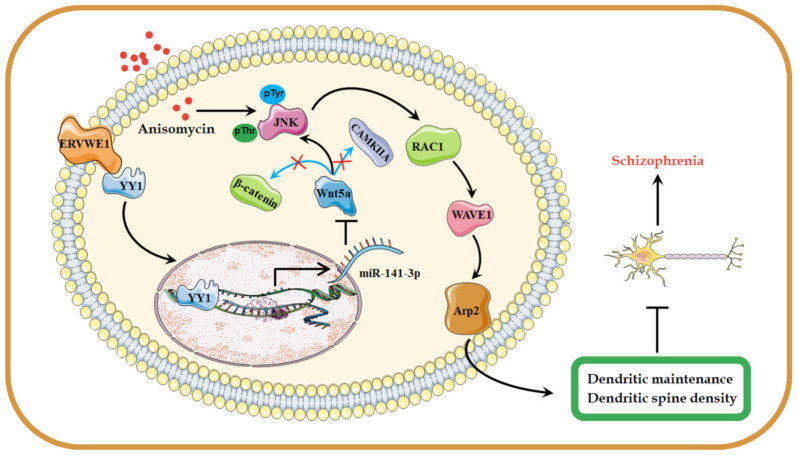
A possible hypothesis that ERVWE1 reduces dendritic spine density and alters dendritic spine morphology. The elevated ERVWE1 increased the expression of miR-141-3p through binding with TF YY1 and inhibited the Wnt/JNK non-canonical signaling pathway, then leading to impaired neuronal morphology and reduced dendritic spine density in rat hippocampal neurons, which played a critical role in the etiopathogenesis of schizophrenia.

We reported an elevated miR-141-3p levels in schizophrenia, which showed a positively association with ERVWE1. Further studies showed that ERVWE1 reduced hippocampal neuron density and impaired dendritic spine morphology through inhibiting Wnt/JNK non-canonical pathway via miR-141-3p, which was considered as the causal related pathogenicity of schizophrenia (Figure 8). This study provided a novel potential blood-based biomarker and new insight into the roles of ERVWE1 in the etiology of schizophrenia.

## Figures and Tables

**Table 1:** Univariate and multivariate analysis of risk factors for schizophrenia.

Characteristics	Univariate (*p*)	Multivariate		
		OR	95% CI	*p*
Gender (female vs. male)	0.755			NA
Age (years)	0.827			NA
Education (years)	0.836			NA
BMI (body mass index)	0.386			NA
Smoking status (yes vs. no)	0.800			NA
miR-141-3p level	0.020	10.716	2.311–49.964	0.002
Wnt5a protein level	<0.001	83.605	82.243–84.989	<0.001
Arp2 protein level	0.001	6.043	5.991–6.095	<0.001
ERVWE1 protein level	<0.001	7.715	7.580–7.852	<0.001

NA: not adopted, OR: odds ratio, CI: confidence interval.

## Data Availability

All data is available from the corresponding author upon request.

## Supplementary Materials

The following supporting information can be downloaded at: https://www.mdpi.com/article/10.3390/v15010168/s1, Table S1: Comparison of blood mRNA samples demographic data between the normal control and recent-onset schizophrenia groups, Table S2**:** Comparison of serum samples demographic data between the normal control and recent-onset schizophrenia groups, Table S3: Primer sequence for cloning and real-time PCR used in this study, Table S4: Antibodies and the dilutions, Table S5**:** The concentration of Wnt5a in the blood of control and schizophrenia patients, Table S6**:** The concentration of RAC1 in the blood of control and schizophrenia patients, Table S7: The concentration of WAVE1 in the blood of control and schizophrenia patients, Table S8: The concentration of Arp2 in the blood of control and schizophrenia patients, Table S9: The concentration of ERVWE1 in the blood of control and schizophrenia patients, Figure S1: The concentration of RAC1 and WAVE1 in the control groups and the schizophrenia patients, Figure S2: The expression of Arp2 in schizophrenia patients according to bioinformatics, Figure S3: The concentration of Wnt5a and Arp2 in the ERVWE1-positive schizophrenia and the ERVWE1-negative schizophrenia, Figure S4: Wnt5a was a target of miR-141-3p, Figure S5: ERVWE1 had no effect on Arp3 expression, nor on Wnt/β-catenin or Wnt/CAMK2A signaling pathways, Figure S6: ERVWE1 and miR-141-3p did not affect the expression of Wnt/β-catenin nor Wnt/CAMK2A signaling pathways.
