# Supplementary material for: ERVWE1 Reduces Hippocampal Neuron Density and Impairs Dendritic Spine Morphology through Inhibiting Wnt/JNK Non-Canonical Pathway via miR-141-3p in Schizophrenia
Source: Viruses. 2023 Jan 5;15(1):168. doi: 10.3390/v15010168 (PMC9863209; doi:10.3390/v15010168)
Supplement: Supplementary file 1 [file viruses-15-00168-s001.zip › viruses-2037169-supplementary.pdf]

Supplementary Materials

ERVWE1 Reduces Hippocampal Neuron Density and Impairs Dendritic Spine Morphology through Inhibiting Wnt/JNK Non-Canonical Pathway via miR-141-3p in Schizophrenia

Wei Yao [1], Ping Zhou [1], Qiujin Yan [1], Xiulin Wu [1], Yaru Xia [1], Wenshi Li [1], Xuhang Li [1], Fan Zhu [1,2,*]

[1] State Key Laboratory of Virology, Department of Medical Microbiology, School of Basic Medical Sciences, Wuhan University, Wuhan 430071, China
[2] Hubei Province Key Laboratory of Allergy & Immunology, Wuhan University, Wuhan 430071, China
* Correspondence: fanzhu@whu.edu.cn or zhufan@hotmail.com

Supplementary Table S1. Comparison of blood mRNA samples demographic data between the normal control and recent-onset schizophrenia groups.

Characteristics	Recent-Onset Schizophrenia (n = 18)	Normal Control (*n* = 25)	*p* Value
Age (years) [a]	43.22 ± 12.99	43.96 ± 12.99	0.971
Education (years) [a]	12.00 ± 3.40	11.36 ± 4.07	0.648
Height [a]	1.67 ± 0.86	1.70 ± 0.88	0.252
BMI (body mass index) [a]	23.32 ± 2.42	22.00 ± 2.73	0.852
Gender (F/M) [b]	8/10	12/13	1.000
Smokers (N, n%) [b]	9(50%)	11(44%)	0.763

Notes: All values are the means ± SD unless otherwise indicated.
[a]: *p* values were calculated by the Mann-Whitney U test.
[b]: *p* values were calculated by the chi-square test.
[c]: In this study, Smokers represents the number of people who have a habit of smoking.

Supplementary Table S2. Comparison of serum samples demographic data between the normal control and recent-onset schizophrenia groups.

Characteristics	Recent-Onset Schizophrenia (n = 48)	Normal Control (*n* = 36)	*p* Value
Age (years) [a]	42.08 ± 12.68	43.92 ± 12.41	0.516
Education (years) [a]	11.54 ± 3.61	11.11 ± 4.12	0.648
Height [a]	1.68 ± 0.08	1.70 ± 0.08	0.224
BMI (body mass index) [a]	21.71 ± 2.72	22.14 ± 2.94	0.493
Gender (F/M) [b]	22/26	17/19	0.899
Smokers (N, n%) [b]	19 (39.58%)	14 (38.89%)	0.949

Notes: All values are the means ± SD unless otherwise indicated.
[a]: *p* values were calculated by the Mann-Whitney U test.
[b]: *p* values were calculated by the chi-square test.
[c]: In this study, Smokers represents the number of people who have a habit of smoking.

Supplementary Table S3. Primer sequence for cloning and real-time PCR used in this study.

Primer	Sequence (5′-3′)
wnt5a-3UTR-F1	ATAGCTAGCCTAAAGGTTCATTGTGTT
wnt5a-3UTR-R1	ATATCTAGAAACTTGGTATTGGCAAAA
wnt5a-mut-R2	GTTTTTGCAATAAAGCGTCTCAGTCAATATACAACTG
wnt5a-mut-F2	CAGTTGTATATTGACTGAGACGCTTTATTGCAAAAAC
YY1-CDS-F	ATACTCGAGATGGCCTCGGGCGACACC
YY1-CDS-R	ATAGGATCCTCACTGGTTGTTTTTGGC
shYY1-F	GATCC GCCCTCATAAAGGCTGCACAA TTCAAGAGA TTGTGCAGCCTTTATGAGGGCA
shYY1-R	AGCTT GCCCTCATAAAGGCTGCACAA TCTCTTGAA TTGTGCAGCCTTTATGAGGGCG
shYY1-NC-F	GATCCCCTTAGTTTATGACCCAATTTCAAGAGAATT-GGGTCATAAACTAAGGA
shYY1-NC-R	AGCTTCCTTAGTTTATGACCCAATTCTCTTGAAATT-GGGTCATAAACTAAGGG
miR141pro-F	ATAGCTAGCAGGGGTGAGACTAGGCAG
miR141pro-R	TATAAGCTTAGATCCCTGGCTCCCATC
hsa-miR-141-3p mimic-F	UAACACUGUCUGGUAAAGAUGG
hsa-miR-141-3p mimic-R	CCATCTTTACCAGACAGTGTTA
mimic-NC-F	UUUGUACUACACAAAAGUACUG
mimic-NC-R	CAGTACTTTTGTGTAGTACAAA
hsa-miR-141-3p inhibitor	CCATCTTTACCAGACAGTGTTA
inhibitor-NC	CAGUACUUUUGUGUAGUACAAA
hsa-miR-129-5p inhibitor	GCAAGCCCAGACCGCAAAAAG
hsa-miR-300 inhibitor	AGAGAGAGTCTGCCCTTGTATA
ERVWE1-F	CCATGCCGCTGTATGACCAG
ERVWE1-R	GGGTTCCCTTAGAAAGACTCCT
GAPDH-F	GGAGCGAGATCCCTCCAAAAT
GAPDH-R	GGCTGTTGTCATACTTCTCATGG
CCNL2-F	GTACTCCGGGGTGCTCATC
CCNL2-R	GAGGTCGGTCTCTGTGTCG
ACOT7-F	TCTCCCATGTGCATCGGTG
ACOT7-R	TTTTCGGACATCACGTTGACC

Supplementary Table S4. Antibodies and the dilutions.

Proteins	Brands	Catalogs	Dilutions
ERVWE1	Abcam	ab179693	1:1000 (WB), 1:100 (IP, IF)
Wnt5a	Abcam	ab229200	1:1000 (WB), 1:100 (IP, IF)
WAVE1	Abcam	ab272916	1:1000 (WB)
RAC1	Abcam	ab180683	1:2000 (WB)
JNK	Abcam	ab179461	1:1000 (WB)
p-JNK (pT183 + Y185)	Abcam	ab179461	1:1000 (WB)
CAMK2A	Abcam	ab92332	1:20000 (WB)
p-CAMK2A (Thr286)	Abcam/ABclonal	ab124880/ AP0255	1:2000 (WB)
Histone H3	Abcam	ab1791	1:2000 (WB)
YY1	Abcam	ab109228	1:5000 (WB)
Arp2	Abcam	ab128934	1:5000 (WB)
MAP2	Abcam	ab32454	1:5000 (WB), 1:100 (IF)
GAPDH	Abcam	ab8245	1:5000 (WB)
Goat anti-rabbit IgG-HRP	Abcam	ab205718	1:10000 (WB)
goat anti-mouse IgG-HRP	Abcam	ab205719	1:10000 (WB)
Cy3 goat Anti-rabbit IgG (H+L)	Abcam	ab6939	1:500 (IF)

Supplementary Table S5. The concentration of Wnt5a in the blood of control and schizophrenia patients.

Control	n	36	Schizophrenia	n	48
	Mean	22023.42		Mean	9186.80
	SE	2043.35		SE	713.30
	Median	18840.93		Median	10264.39
	SD	12260.12		SD	4941.88
	Minimum	2123.92		Minimum	644.61
	Maximum	57874.52		Maximum	18986.01
	Range	55750.60		Range	18341.40
	IQR	18499.99		IQR	9395.42
	Skewness	0.52		Skewness	-0.093
	Kurtosis	0.45		Kurtosis	-1.15

SD: Standard deviation; SE: Standard error, IQR: Interquartile range.

Supplementary Table S6. The concentration of RAC1 in the blood of control and schizophrenia patients.

Control	n	36	Schizophrenia	n	48
	Mean	42800.44		Mean	19779.49
	SE	7324.85		SE	6306.64
	Median	36520.65		Median	7715.00
	SD	43949.12		SD	43693.68
	Minimum	4415.80		Minimum	2732.80
	Maximum	284963.00		Maximum	232698.00
	Range	280547.20		Range	229965.20
	IQR	8411.00		IQR	4266.95
	Skewness	5.01		Skewness	4.26
	Kurtosis	28.07		Kurtosis	18.12

SD: Standard deviation; SE: Standard error, IQR: Interquartile range.

Supplementary Table S7. The concentration of WAVE1 in the blood of control and schizophrenia patients.

Control	n	36	Schizophrenia	n	48
	Mean	136152.09		Mean	67856.49
	SE	13745.44		SE	20321.71
	Median	131440.50		Median	25435.05
	SD	82472.64		SD	140792.91
	Minimum	9528.40		Minimum	4284.30
	Maximum	472233.50		Maximum	759105.20
	Range	462705.10		Range	754820.90
	IQR	67356.15		IQR	22916.93
	Skewness	1.72		Skewness	3.83
	Kurtosis	7.05		Kurtosis	14.89

SD: Standard deviation; SE: Standard error, IQR: Interquartile range.

Supplementary Table S8. The concentration of Arp2 in the blood of control and schizophrenia patients.

Control	*n*	36	Schizophrenia	*n*	48
	Mean	7216.01		Mean	5909.18
	SE	401.43		SE	150.27
	Median	8271.68		Median	5994.48
	SD	2408.58		SD	1041.07
	Minimum	681.06		Minimum	3457.94
	Maximum	9978.75		Maximum	8008.11
	Range	9297.69		Range	4550.17
	IQR	3259.16		IQR	1434.71
	Skewness	-1.25		Skewness	-0.21
	Kurtosis	0.55		Kurtosis	-0.35

SD: Standard deviation; SE: Standard error, IQR: Interquartile range.

Supplementary Table S9. The concentration of ERVWE1 in the blood of control and schizophrenia patients.

Control	*n*	36	Schizophrenia	*n*	48
	Mean	1436.67		Mean	8986.73
	SE	275.66		SE	1581.54
	Median	960.82		Median	4764.14
	SD	1653.97		SD	10957.22
	Minimum	629.18		Minimum	864.21
	Maximum	10166.55		Maximum	37913.18
	Range	9537.37		Range	37048.97
	IQR	551.00		IQR	5982.00
	Skewness	4.59		Skewness	1.69
	Kurtosis	23.40		Kurtosis	1.43

SD: Standard deviation; SE: Standard error, IQR: Interquartile range.

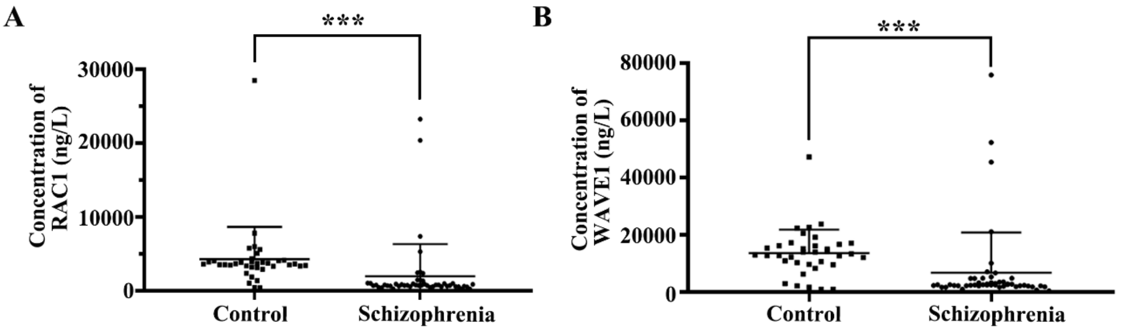

Supplementary Figure S1. (**A, B**) The concentration of RAC1 and WAVE1 in the control groups (n = 36) and the schizophrenia patients (n = 48). *** p<0.001.

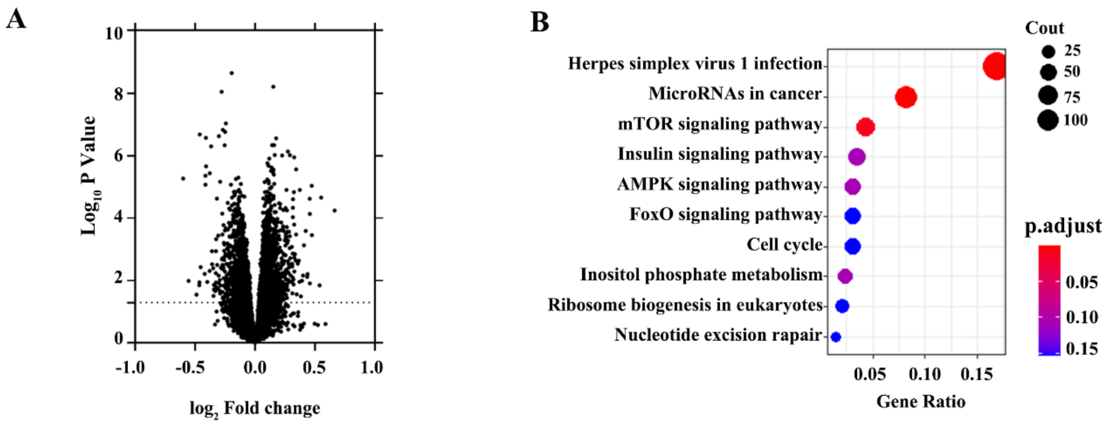

Supplementary Figure S2. The expression of Arp2 in schizophrenia patients according to bioinformatics. (**A**) The volcano plot of gene expression profile data and 255 differentially expressed genes identified using R software. (**B**) GO, and KEGG analyses of the differentially expressed genes indicated enrichment in the herpes simplex virus I infection, microRNAs in cancer, mTOR signaling pathway, etc.

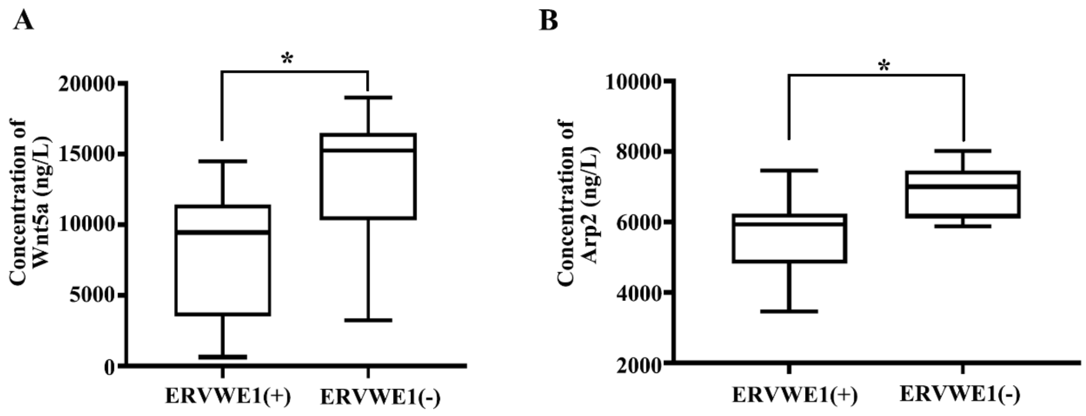

Supplementary Figure S3 (**A, B**) The concentration of Wnt5a and Arp2 in the ERVWE1-positive schizophrenia (ERVWE1 (+), n = 37) and the ERVWE1-negative schizophrenia (ERVWE1 (-), n = 11) groups., * p<0.05.

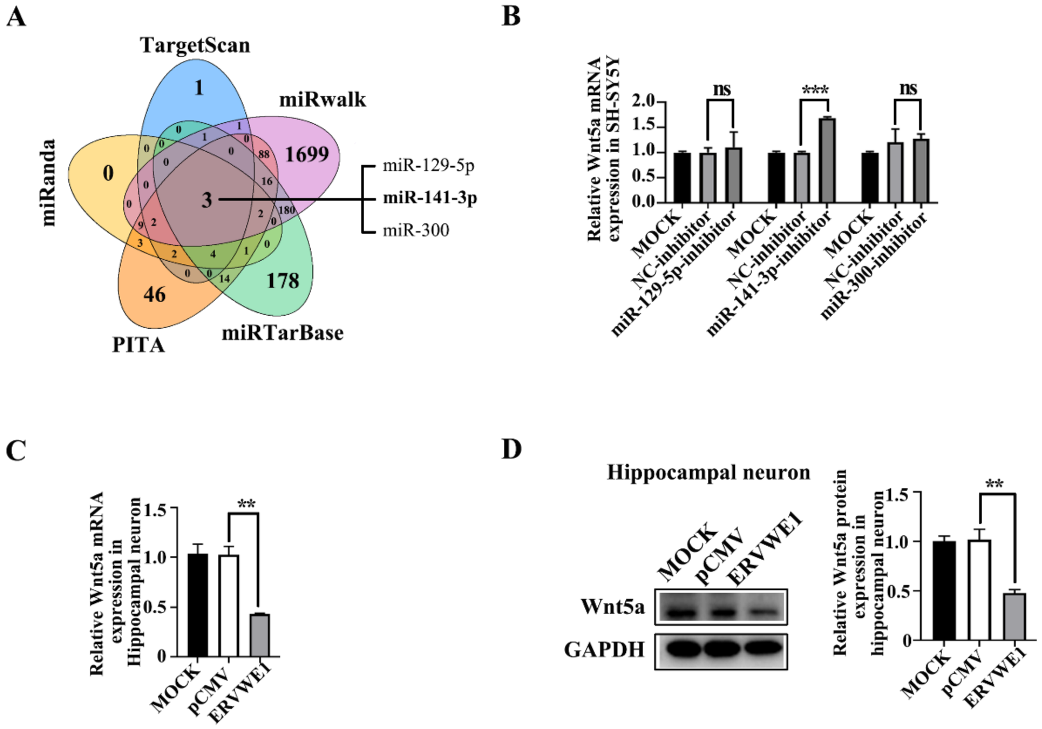

Supplementary Figure S4. Wnt5a was a target of miR-141-3p. (**A**) Targetscan, miRanda, PITA, miRTarBase and miRwalk were used to predict the miRNA regulating Wnt5a, the results showed that miR-129-5p, miR-141-3p, and miR-300 might regulate the expression of Wnt5a. (**B**) Knockout of miR-141-3p increased the mRNA level of Wnt5a. (**C**) Decreased mRNA level of Wnt5a was detected using RT-qPCR in rat hippocampal neurons. (**D**) ERVWE1 down-regulated the protein level of Wnt5a in rat hippocampal neurons. ***p<0.001, **p<0.01, ns p>0.05.

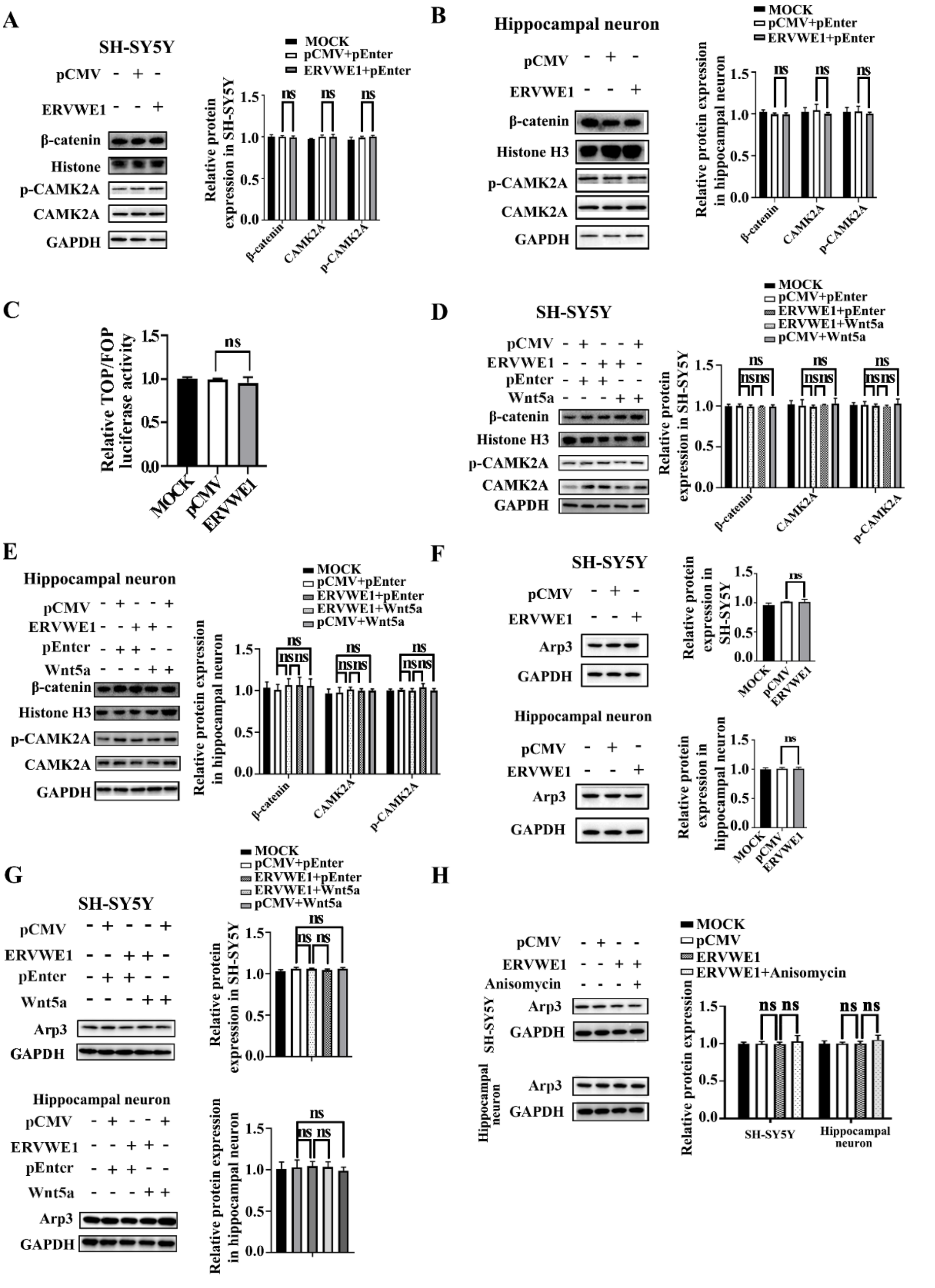

Supplementary Figure S5. ERVWE1 had no effect on Arp3 expression, nor on Wnt/β-catenin or Wnt/CAMK2A signaling pathways. (**A, B**) ERVWE1 did not affect the expression of β-catenin, Histone H3, p-CAMK2A, and CAMK2A in SH-SY5Y cells and rat hippocampal neurons. (**C**) ERVWE1 had no effect on TOP/FOP reporter activity in SH-SY5Y cells with ERVWE1. A renilla transfection control normalized all results. (**D, E**) The levels of β-catenin, Histone H3, p-CAMK2A, CAMK2A had no significant change in SH-SY5Y cells and rat hippocampal neurons. (**F**) No significant changes were noted in the protein level of Arp3 in SH-SY5Y cells and rat hippocampal neurons. (**G**) The levels of Arp3 had no significant change in SH-SY5Y cells and rat hippocampal neurons. (H) SH-SY5Y cells and rat hippocampal neurons were co-transfected with ERVWE1 and the JNK activator anisomycin, and the Arp3 protein had no significant change. ns $p>0.05$.

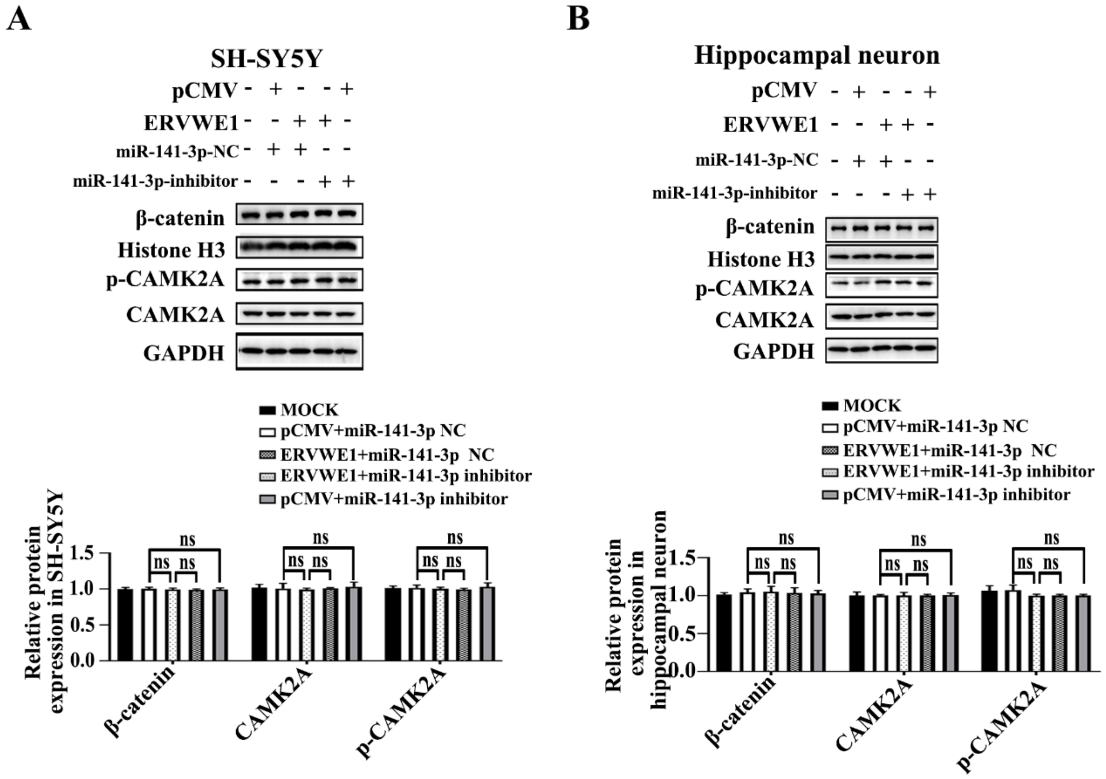

Supplementary Figure S6. ERVWE1 and miR-141-3p did not affect the expression of Wnt/β-catenin nor Wnt/CAMK2A signaling pathways. (**A, B**) SH-SY5Y cells and hippocampus neurons were co-transfected with ERVWE1, miR-141-3p inhibitor or their corresponding controls, and the levels of β-catenin, Histone H3, p-CAMK2A, CAMK2A were unchanged. ns p>0.05.
